# Thirty complete *Streptomyces* genome sequences for mining novel secondary metabolite biosynthetic gene clusters

**DOI:** 10.1038/s41597-020-0395-9

**Published:** 2020-02-13

**Authors:** Namil Lee, Woori Kim, Soonkyu Hwang, Yongjae Lee, Suhyung Cho, Bernhard Palsson, Byung-Kwan Cho

**Affiliations:** 1 Department of Biological Sciences and KI for the BioCentury, Korea Advanced Institute of Science and Technology, Daejeon, 34141 Republic of Korea; 2 Intelligent Synthetic Biology Center, Daejeon, 34141 Republic of Korea; 3 Department of Bioengineering, University of California San Diego, La Jolla, CA 92093 USA; 4 Department of Pediatrics, University of California San Diego, La Jolla, CA 92093 USA; 5 Novo Nordisk Foundation Center for Biosustainability, Technical University of Denmark, Lyngby, 2800 Denmark

**Keywords:** Genomic analysis, Systems biology, Antibiotics

## Abstract

*Streptomyces* are Gram-positive bacteria of significant industrial importance due to their ability to produce a wide range of antibiotics and bioactive secondary metabolites. Recent advances in genome mining have revealed that *Streptomyces* genomes possess a large number of unexplored silent secondary metabolite biosynthetic gene clusters (smBGCs). This indicates that *Streptomyces* genomes continue to be an invaluable source for new drug discovery. Here, we present high-quality genome sequences of 22 *Streptomyces* species and eight different *Streptomyces venezuelae* strains assembled by a hybrid strategy exploiting both long-read and short-read genome sequencing methods. The assembled genomes have more than 97.4% gene space completeness and total lengths ranging from 6.7 to 10.1 Mbp. Their annotation identified 7,000 protein coding genes, 20 rRNAs, and 68 tRNAs on average. *In silico* prediction of smBGCs identified a total of 922 clusters, including many clusters whose products are unknown. We anticipate that the availability of these genomes will accelerate discovery of novel secondary metabolites from *Streptomyces* and elucidate complex smBGC regulation.

## Abstract (summary)

**Table:** 

Measurement(s)	DNA • genome • sequence_assembly • sequence feature annotation
Technology Type(s)	DNA sequencing • sequence assembly process • sequence annotation
Factor Type(s)	strain
Sample Characteristic - Organism	Streptomyces

Machine-accessible metadata file describing the reported data: 10.6084/m9.figshare.11791323

## Background & Summary

With the rapid emergence of antibiotic microbial resistance (AMR) to all major classes of antibiotics and the decline in number of potential candidates for new antibiotics, there is a pressing need for the discovery of novel antibacterial compounds^1^. *Streptomyces*, soil dwelling gram-positive bacteria, continue to be promising microorganisms for the production of clinically important secondary metabolites, including not only antibiotics, but also antiviral, antifungal, and antiparasitic agents, and antitumorals and immunosuppressant compounds^2^. *Streptomyces* are distinguished by their complex life cycle and high G + C content (often over 70%) in their linear genomes^3,4^. Traditionally, drug discovery from *Streptomyces* has been based on bioactivity screening followed by mass spectrometry and NMR-based molecular identification^5^. However, recent advances in genomics-based approaches revealed that most of the secondary metabolite biosynthetic gene clusters (smBGCs) of streptomycetes are inactive under laboratory conditions, suggesting that the ability of streptomycetes to produce secondary metabolites has been under-estimated^5,6^. Each *Streptomyces* species has the genetic potential to produce more than 30 secondary metabolites on average, which are diverse and differ between species^7,8^. Considering *Streptomyces* is the largest genus of actinobacteria with approximately 900 species characterized so far, streptomycetes are a valuable resource for the discovery of novel secondary metabolites^9^.

SmBGCs, especially polyketide and non-ribosomal peptide synthetase types, are often composed of extraordinarily long genes (>5 kb) encoding multi-modular enzymes with repetitive domain structures. Therefore, accurate gene annotations based on high quality genome sequences are essential for the precise identification of smBGCs^10^. Gene annotation with the high quality genome of *S. clavuligerus* revealed that 30% out of a total of 7,163 protein coding genes were incorrectly annotated in the previous draft genome of *S. clavuligerus* containing ambiguous and inaccurate nucleotides, indicating the importance of high quality genome sequences^11^. In addition, high quality genome sequences are essential for multi-omics analysis, which facilitates the understanding of the complex regulation on smBGCs and rational engineering for increasing secondary metabolites production^11,12^.

Among the 1,614 streptomycetes genomes that have been deposited in the NCBI Assembly database to date (as of 9th December 2019), only 189 and 35 assemblies were designated as complete genome level and chromosome level, respectively. More than 86% of assemblies were draft-quality genome sequences, which contain fragmented multiple contigs or ambiguous sequences^4,13–15^. One of the main obstacles to obtaining high quality genomic information of streptomycetes is the low fidelity of sequencing techniques when dealing with high G w C genomes and frequently repetitive sequences such as terminal inverted repeats^13^. In addition, since streptomycetes have linear chromosome, it is difficult to confirm the completeness of the assembled chromosome.

In this study, we present the high-quality genome sequences of 30 streptomycetes, increasing the total number of reported complete *Streptomyces* genome by about 10%. The target streptomycetes were 22 *Streptomyces* type strains and eight different *Streptomyces venezuelae* strains, most of which are currently used as industrial strains for producing various bioactive compounds. We applied hybrid assembly strategy with long-read (PacBio) and short-read (Illumina) sequencing techniques to obtain complete genome sequences. PacBio sequencing provides long reads of several kb in length which allows the readthrough of regions with low complexity, enabling the assembly of repetitive regions, which are difficult to assemble by using Illumina sequencing reads, even with the high coverage data^16^. However, Illumina sequencing provides reads with a lower error rate compared to the PacBio sequencing, and assembled contigs based on the Illumina sequencing reads are not simply a subset of the contigs from PacBio sequencing reads^13,17^. Therefore, reconciling PacBio and Illumina sequencing methods enables one to generate more complete genomes by overcoming the shortcomings of each method. During the genome assembly using reads from PacBio (0.46~5.18 Gbp) and Illumina (0.5~3.0 Gbp) sequencing, we constructed 6.7 to 10.1 Mbp of streptomycetes genomes, most of which consist of single chromosomes with 72% G + C contents on average. Inaccurate sequences in the assembled genome were corrected using Illumina sequencing reads. The complete streptomycetes genomes have more than 97.4% gene space completeness and on average 7,000 protein coding genes, 20 rRNAs, and 68 tRNAs were annotated. Finally, based on the complete genome sequences and annotations, we predicted a total of 922 smBGCs. The complete genome sequences and newly determined smBGCs in this study should prove to be a fundamental resource for understanding the genetic basis of streptomycetes and for discovering novel secondary metabolites.

## Methods

### Genomic DNA (gDNA) extraction

Total 30 streptomycetes were purchased from Korean Collection for Type Cultures (KCTC, Korea). A stock of streptomycetes were inoculated to 50 mL of liquid culture medium with 0.16 g mL^−1^ of glass beads (3 ± 0.3 mm diameter) in 250 mL baffled flask and grown at 30 °C in a 200 rpm orbital shaker. Each streptomycetes was grown in one of four different culture medium, R5(–) medium (25 mM TES (pH 7.2), 103 g L^−1^ sucrose, 1% (w/v) glucose, 5 g L^−1^ yeast extract, 10.12 g L^−1^ MgCl_2_∙6H_2_O, 0.25 g L^−1^ K_2_SO_4_, 0.1 g L^−1^ casamino acids, 0.08 g L^−1^ ZnCl_2_, 0.4 mg L^−1^ FeCl_3_, 0.02 mg L^−1^ CuCl_2_∙2H_2_O, 0.02 mg L^−1^ MnCl_2_∙4H_2_O, 0.02 mg L^−1^ Na_2_B_4_O_7_∙10H_2_O, and 0.02 mg L^−1^ (NH_4_)_6_Mo_7_O_24_∙4H_2_O), 1 × sporulation medium (3.33 g L^−1^ glucose, 1 g L^−1^ yeast extract, 1 g L^−1^ beef extract, 2 g L^−1^ tryptose, and 0.006 g L^−1^ FeSO_4_∙7H_2_O), YEME medium (340 g L^−1^ sucrose, 10 g L^−1^ glucose, 3 g L^−1^ yeast extract, 5 g L^−1^ bacto peptone, and 3 g L^−1^ oxoid malt extract), and MYM medium (4 g L^−1^ maltose, 4 g L^−1^ yeast extract, 10 g L^−1^ malt extract). For gDNA extraction, 25 mL cultured cells were harvested at the exponential growth phase and washed twice with same volume of 10 mM EDTA, followed by the lysozyme (10 mg mL^−1^) treatment at 37 °C for 45 min. gDNA was extracted using a Wizard Genomic DNA Purification Kit (Promega, Madison, WI, USA) according to the manufacturer’s instruction. Quality and quantity of extracted gDNA samples were evaluated using 1% agarose gel electrophoresis and Nanodrop (Thermo Fisher Scientific, Waltham, MA, USA), respectively.

### Short-read (Illumina) genome sequencing

**Fig. 1:**
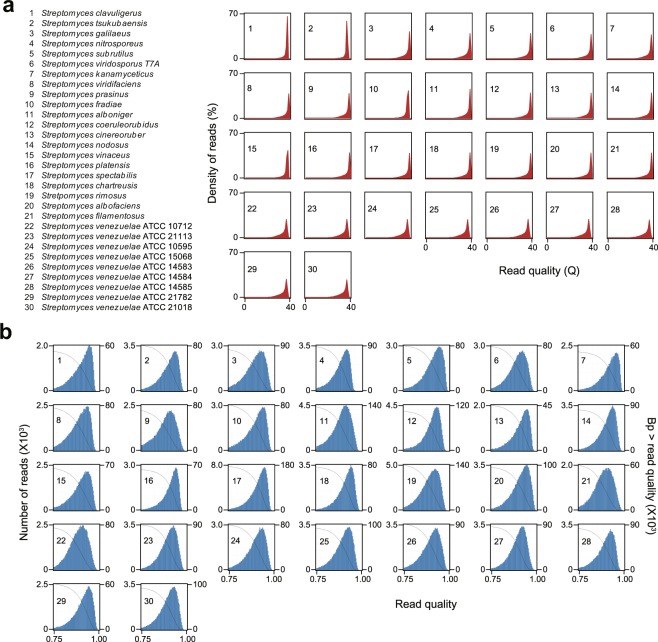
Quality of the genome sequencing data. (**a**) Distribution of Illumina reads quality based on Phred score. (**b**) Read quality distribution of PacBio reads. Black line indicates total number of bases in the reads which have greater read quality than the corresponding read quality value on x-axis.

For construction of short-read genome sequencing library, 2.5 μg of gDNA was sheared to approximately 350 bp by a Covaris instrument (Covaris Inc., Woburn, MA, USA) with the following conditions; Power 175, Duty factor 20%, C. burst 200, Time 23 s, 8 times. The library was constructed using a TruSeq DNA PCR-Free LT kit (Illumina Inc., San Diego, CA, USA) following manufacturer’s instruction. Briefly, the fragmented DNA samples were cleaned and end-repaired, followed by the adaptor ligation and bead-based size selection ranging from 400 to 500 bp. Quantity of final libraries was measured using Qubit® dsDNA HS Assay Kit (Thermo Fisher Scientific) and the library size was determined using Agilent 2200 Bioanalyzer (Agilent Technologies, Santa Clara, CA, USA). Among the constructed sequencing libraries, 29 libraries were sequenced with the HiSeq. 2500 (Illumina Inc.) as 100 bp single-end reads and remaining one library for *S. tsukubaensis* was sequenced with the Miseq v.2 (Illumina Inc.) with 50 bp single-read recipe. Finally, 0.46 to 5.18 Gbp of raw sequence data were obtained and the read qualities were examined by creating sequencing QC reports function of CLC genomic workbench version 6.5.1 (CLC bio, Denmark) (Online-only Table 1 and Fig. 1a).

### Long-read (PacBio) genome sequencing

A total of 5 μg gDNA was used as input for PacBio genome sequencing library preparation. The sequencing library was constructed with the PacBio SMRTbell^TM^ Template Prep Kit (Pacific Biosciences, Menlo Park, CA, USA) following manufacturer’s instructions. Fragments smaller than 20 kbp were removed using the Blue Pippin Size selection system (Sage Science, Beverly, MA, USA) and the constructed libraries were validated using Agilent 2100 Bioanalyzer (Agilent Technologies). Final SMRTbell libraries were sequenced using one or two SMRT cells with P6-C4-chemistry (DNA Sequencing Reagent 4.0) on the PacBio RS II sequencing platform (Pacific Biosciences). Approximately, 0.5 to 3.0 Gbp of raw sequence data were generated (Online-only Table 1).

### Genome assembly

Among the raw PacBio sequencing reads, only the reads with a read quality value greater than 0.75 and a length longer than 50 bp were filtered (Fig. 1b). Post filtered reads were assembled by the hierarchical genome assembly process workflow (HGAP, Version 2.3), including consensus polishing with Quiver^18^. For each assembled contig, error correction was performed based on their estimated genome size and average coverage. Raw reads from the Illumina sequencing were quality trimmed using CLC genomic workbench version 6.5.1 (ambiguous limit 2 and quality limit 0.05) and assembled using *de novo* assembly function of CLC genomic workbench version 6.5.1 with default parameters. To expand the assembled contigs, all of assembled PacBio and Illumina contigs were aligned using MAUVE 2.4.0^19^ and linked using GAP5 program (Staden package)^20^.

### Genome correction

**Fig. 2:**
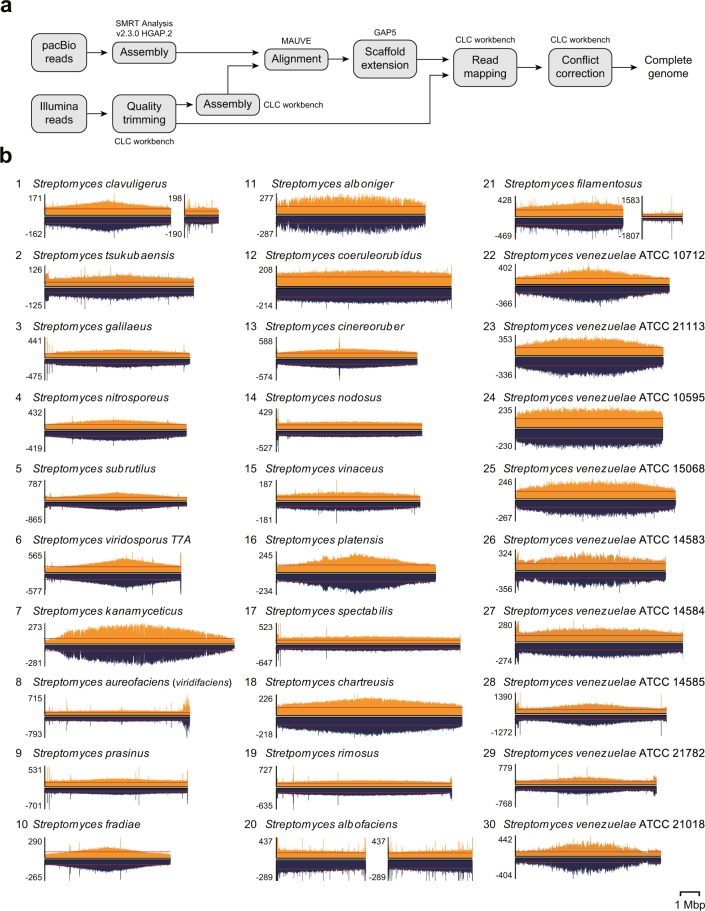
Genome assembly of 30 streptomycetes. (**a**) Strategy for genome assembly and corrections. (**b**) Profile of Illumina reads mapped on assembled genomes. Data were visualized using SignalMap (Roche NimbleGen, Inc.). Red line indicates the average Illumina read coverage of all genomic positions.

Quality trimmed Illumina sequencing reads were mapped to the assembled genome using CLC genomic workbench version 6.5.1 (mismatch cost 2, insertion cost 3, deletion cost 3, length fraction 0.9, and similarity fraction 0.9). Conflicts showing more than 80% frequency for Illumina reads were corrected as Illumina sequence (Table 1). In addition, percentage of mapped Illumina reads on to the assembled genome represents degree of completeness (Table 1 and Fig. 2b). Completeness of gene space was estimated using the BUSCO v3 (Table 2)^21^.

**Table 1:** The statistics of genome assembly and correction.

No.	Species	Final scaffolds (No.)	Scaffold length before correction (bp)	Mapped Illumina reads (%)	Conflict positions (No.)	Added bases (No.)	Deleted bases (No.)	Scaffold length after correction (bp)	G + C contets (%)	Assembly accession number
1	*Streptomyces clavuligerus*	2	6,748,589 and 1,795,496	71.16 and 14.03	7	4	3	6,748,591 and 1,795,495	72.5	GCA_005519465.1
2	*Streptomyces tsukubaensis*	1	7,963,727	95.13	15	15	0	7,963,742	71.9	GCA_003932715.1
3	*Streptomyces galilaeus*	1	7,756,176	90.56	51	34	16	7,756,194	71.4	GCA_008704575.1
4	*Streptomyces nitrosporeus*	1	7,581,543	93.50	51	35	16	7,581,562	72.2	GCA_008704555.1
5	*Streptomyces subrutilus*	1	7,604,705	96.41	286	269	0	7,604,974	73.4	GCA_008704535.1
6	*Streptomyces viridosporus* T7A	1	7,280,447	90.44	90	89	0	7,280,536	72.6	GCA_008704515.1
7	*Streptomyces kanamyceticus*	1	10,133,525	99.09	376	375	3	10,133,897	71.0	GCA_008704495.1
8	*Streptomyces aureofaciens*	1	7,757,873	84.86	16	9	5	7,757,877	72.6	GCA_008704475.1
9	*Streptomyces prasinus*	1	7,646,576	89.70	1,025	1,021	5	7,647,592	72.0	GCA_008704445.1
10	*Streptomyces fradiae*	1	6,725,574	97.63	5	5	0	6,725,579	74.7	GCA_008704425.1
11	*Streptomyces alboniger*	1	7,962,594	99.12	193	193	1	7,962,786	71.2	GCA_008704395.1
12	*Streptomyces coeruleorubidus*	1	9,334,399	99.67	1,297	1,299	0	9,335,698	71.1	GCA_008705135.1
13	*Streptomyces cinereoruber*	1	7,516,474	99.74	178	178	0	7,516,652	72.9	GCA_009299385.1
14	*Streptomyces nodosus*	1	7,772,564	99.51	26	25	2	7,772,587	70.9	GCA_008704995.1
15	*Streptomyces vinaceus*	1	7,673,329	92.46	180	180	0	7,673,509	72.3	GCA_008704935.1
16	*Streptomyces platensis*	1	8,500,673	99.75	354	352	13	8,501,012	71.1	GCA_008704855.1
17	*Streptomyces spectabilis*	1	9,806,222	95.30	934	938	0	9,807,160	72.4	GCA_008704795.1
18	*Streptomyces chartreusis*	1	9,911,637	98.42	461	461	0	9,912,098	71.0	GCA_008704715.1
19	*Stretpomyces rimosus*	1	9,361,132	96.22	22	22	0	9,361,154	72.0	GCA_008704655.1
20	*Streptomyces albofaciens*	2	4,757,761 and 4,494,336	53.36 and 45.53	504	501	3	4,757,978 and 4,494,617	72.3	GCA_008634025.1
21	*Streptomyces filamentosus*	2	5,742,252 and 2,129,928	75.22 and 24.28	3,218	3,228	1	5,744,022 and 2,131,385	73.6	GCA_008634015.1
22	*Streptomyces venezuelae* ATCC 10712	1	8,223,439	99.84	96	81	15	8,223,505	72.5	GCA_008639165.1
23	*Streptomyces venezuelae* ATCC 21113	1	7,893,622	99.85	173	181	0	7,893,803	72.5	GCA_008639045.1
24	*Streptomyces venezuelae* ATCC 10595	1	7,871,449	95.50	35	34	3	7,871,480	72.5	GCA_008705255.1
25	*Streptomyces venezuelae* ATCC 15068	1	8,557,615	99.71	587	587	0	8,558,202	71.9	GCA_008642375.1
26	*Streptomyces venezuelae* ATCC 14583	1	8,018,461	87.17	29	27	4	8,018,484	71.3	GCA_008642355.1
27	*Streptomyces venezuelae* ATCC 14584	1	8,941,823	99.00	255	255	0	8,942,078	71.2	GCA_008642315.1
28	*Streptomyces venezuelae* ATCC 14585	1	8,048,139	82.34	64	41	26	8,048,154	71.3	GCA_008642335.1
29	*Streptomyces venezuelae* ATCC 21782	1	7,525,235	90.50	87	87	0	7,525,322	71.9	GCA_008642295.1
30	*Streptomyces venezuelae* ATCC 21018	1	7,746,214	91.61	59	57	4	7,746,267	72.1	GCA_008642275.1

**Table 2:** Gene space completeness of completed genomes.

No.	Species	Complete and single-copy	Complete and duplicated	Fragmented	Missing	Total	Gene space completeness (%)
1	*Streptomyces clavuligerus*	343	0	0	9	352	97.4
2	*Streptomyces tsukubaensis*	350	0	0	2	352	99.4
3	*Streptomyces galilaeus*	351	0	0	1	352	99.7
4	*Streptomyces nitrosporeus*	352	0	0	0	352	100.0
5	*Streptomyces subrutilus*	349	0	0	3	352	99.1
6	*Streptomyces viridosporus* T7A	351	0	0	1	352	99.7
7	*Streptomyces kanamyceticus*	352	0	0	0	352	100.0
8	*Streptomyces aureofaciens*	350	0	0	2	352	99.4
9	*Streptomyces prasinus*	350	0	0	2	352	99.4
10	*Streptomyces fradiae*	351	0	0	1	352	99.7
11	*Streptomyces alboniger*	351	0	0	1	352	99.7
12	*Streptomyces coeruleorubidus*	351	0	0	1	352	99.7
13	*Streptomyces cinereoruber*	351	0	0	1	352	99.7
14	*Streptomyces nodosus*	350	0	1	1	352	99.4
15	*Streptomyces vinaceus*	349	0	1	2	352	99.1
16	*Streptomyces platensis*	351	0	0	1	352	99.7
17	*Streptomyces spectabilis*	350	0	1	1	352	99.4
18	*Streptomyces chartreusis*	351	0	0	1	352	99.7
19	*Stretpomyces rimosus*	351	0	0	1	352	99.7
20	*Streptomyces albofaciens*	346	4	0	2	352	99.4
21	*Streptomyces filamentosus*	351	0	0	1	352	99.7
22	*Streptomyces venezuelae* ATCC 10712	352	0	0	0	352	100.0
23	*Streptomyces venezuelae* ATCC 21113	352	0	0	0	352	100.0
24	*Streptomyces venezuelae* ATCC 10595	352	0	0	0	352	100.0
25	*Streptomyces venezuelae* ATCC 15068	351	0	0	1	352	99.7
26	*Streptomyces venezuelae* ATCC 14583	351	0	0	1	352	99.7
27	*Streptomyces venezuelae* ATCC 14584	351	0	0	1	352	99.7
28	*Streptomyces venezuelae* ATCC 14585	351	0	0	1	352	99.7
29	*Streptomyces venezuelae* ATCC 21782	349	0	0	3	352	99.1
30	*Streptomyces venezuelae* ATCC 21018	350	0	0	2	352	99.4

### Genome annotation and secondary metabolite biosynthetic gene cluster prediction

The complete genome sequences of streptomycetes were submitted to the NCBI GenBank database and annotated by the latest updated version of NCBI Prokaryotic Genome Annotation Pipeline (PGAP)^22^. Using the GenBank formatted files of each genomes as input, secondary metabolite biosynthetic gene clusters were predicted by antiSMASH 4.0^23^.

## Data Records

Raw reads from short-read (Illumina) and long-read (PacBio) sequencing were deposited in the NCBI Sequence Read Archive (SRA) (Online-only Table 1)^24,25^. 30 complete genome sequences were deposited in GenBank via the NCBI’s submission portal (Table 3)^26–55^. Detailed information on the predicted 922 smBGCs in 30 streptomycetes genomes has been deposited in FigShare^56^.

**Table 3:** Summary of genome annotation.

No.	Species	CDS (No.)	16s rRNA (No.)	tRNA (No.)	Genome accession number	BioProject accession number
1	*Streptomyces clavuligerus*	6,880	18	66	CP027858	PRJNA414136
2	*Streptomyces tsukubaensis*	6,376	18	66	CP020700	PRJNA382016
3	*Streptomyces galilaeus*	6,725	18	76	CP023703	PRJNA412292
4	*Streptomyces nitrosporeus*	6,364	18	74	CP023702	PRJNA412292
5	*Streptomyces subrutilus*	6,431	21	68	CP023701	PRJNA412292
6	*Streptomyces viridosporus* T7A	6,211	18	70	CP023700	PRJNA412292
7	*Streptomyces kanamyceticus*	8,384	18	66	CP023699	PRJNA412292
8	*Streptomyces aureofaciens*	6,453	33	71	CP023698	PRJNA412292
9	*Streptomyces prasinus*	6,263	18	68	CP023697	PRJNA412292
10	*Streptomyces fradiae*	5,465	18	65	CP023696	PRJNA412292
11	*Streptomyces alboniger*	6,613	18	67	CP023695	PRJNA412292
12	*Streptomyces coeruleorubidus*	8,058	18	67	CP023694	PRJNA412292
13	*Streptomyces cinereoruber*	6,392	18	69	CP023693	PRJNA412292
14	*Streptomyces nodosus*	6,491	18	68	CP023747	PRJNA412292
15	*Streptomyces vinaceus*	6,603	21	68	CP023692	PRJNA412292
16	*Streptomyces platensis*	7,032	21	67	CP023691	PRJNA412292
17	*Streptomyces spectabilis*	8,212	18	65	CP023690	PRJNA412292
18	*Streptomyces chartreusis*	8,396	18	71	CP023689	PRJNA412292
19	*Stretpomyces rimosus*	7,756	21	68	CP023688	PRJNA412292
20	*Streptomyces albofaciens*	7,520	21	67	PDCM00000000	PRJNA412292
21	*Streptomyces filamentosus*	6,832	24	70	PDCL00000000	PRJNA412292
22	*Streptomyces venezuelae* ATCC 10712	7,377	21	67	CP029197	PRJNA454547
23	*Streptomyces venezuelae* ATCC 21113	6,987	21	67	CP029196	PRJNA454547
24	*Streptomyces venezuelae* ATCC 10595	6,942	21	67	CP029195	PRJNA454547
25	*Streptomyces venezuelae* ATCC 15068	7,700	21	69	CP029194	PRJNA454547
26	*Streptomyces venezuelae* ATCC 14583	7,154	18	66	CP029193	PRJNA454547
27	*Streptomyces venezuelae* ATCC 14584	7,832	18	65	CP029192	PRJNA454547
28	*Streptomyces venezuelae* ATCC 14585	7,096	18	66	CP029191	PRJNA454547
29	*Streptomyces venezuelae* ATCC 21782	6,655	18	69	CP029190	PRJNA454547
30	*Streptomyces venezuelae* ATCC 21018	6,769	21	71	CP029189	PRJNA454547

## Technical Validation

**Fig. 3:**
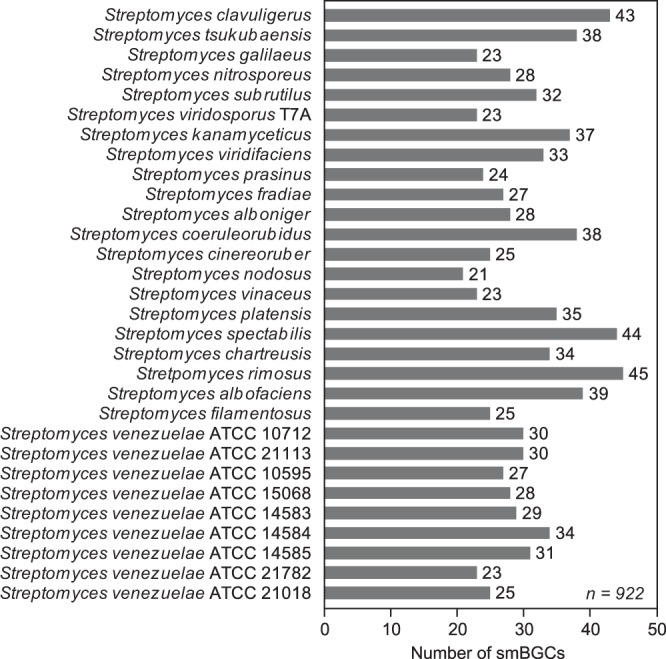
Secondary metabolite biosynthetic gene clusters in 30 complete streptomycetes genomes.

*Streptomyces* have drawn considerable attention because of their ability to produce various clinically important secondary metabolites. Total 30 streptomycetes genomes were sequenced by using both PacBio and Illumina sequencing methods to elucidate their biosynthetic potential. After cleaning the reads, on average 98,380 PacBio reads with 11,725 bp length and 18,223,235 Illumina reads with 100 bp length (50 bp for *S. tsukubaensis*) were generated (Fig. 1a,b and Online-only Table 1). Through the assembly of reads from two sequencing platforms using HGAP, CLC workbench, MAUVE, and GAP5 programs, single linear scaffolds ranging from 6.7 to 10.1 Mbp in length with 72% G + C contents were obtained for 27 streptomycetes, whereas two scaffolds were finally constructed for three remaining streptomycetes, *S. clavuligerus* (6.7 and 1.8 Mbp), *S. albofaciens* (4.8 and 4.5 Mbp), and *S. filamentosus* (5.7 and 2.1 Mbp) (Table 1). *S. clavuligerus* has been reported to have a large linear plasmid with a length of 1.8 Mbp, so the genome was correctly assembled into a single chromosome, while the *S. albofaciens* and *S. filamentosus* genomes appear to be assembled into two divided scaffolds^11,57^. To increase the accuracy of the assembled genome sequences, Illumina sequences showing more than 80% coverage at the conflict sites were taken as the corrected ones (Table 1). Approximately, 96.32% of Illumina sequencing reads were successfully mapped to the corresponding genomes (Table 1 and Fig. 2b). The completeness of the genomes were assessed using the BUSCO approach with a total of 352 orthologue groups from the Actinobacteria Dataset^21^. Results showed that 29 genomes have more than 99.1% gene space completeness and the *S. clavuligerus* genome has 97.4% gene space completeness (Table 2). Following NCBI PGAP, 30 genomes were annotated with 7,000 protein coding genes, 20 rRNAs, and 68 tRNAs on average (Table 3). Finally, based on the annotation, a total of 922 smBGCs were predicted in 30 streptomycetes genomes (Fig. 3). Detailed information, such as genomic positions, types, and putative products of each smBGC are publicly available in Figshare^56^.

## Data Availability

The version and parameter of all bioinformatics tools used in this work are described in the Methods section.

## Online-only Table

**Online-only Table 1:** Summary of PacBio and Illumina genome sequencing data for 30 streptomycetes.

No.	Species	Strain	Platform	Raw reads (No.)	Mean raw reads length (bp)	Clean reads (No.)	Mean clean reads length (bp)	SRA accession number
1	*Streptomyces clavuligerus*	ATCC27064	Illumina	9,853,205	100	9,852,872	100	SRR9192366
			pacBio	150,292	6,572	60,834	15,473	SRR9290551
2	*Streptomyces tsukubaensis*	NRRL18488	Illumina	9,181,843	50	9,176,299	50	SRR9192343
			pacBio	150,292	8,163	93,481	12,715	SRR9290550
3	*Streptomyces galilaeus*	ATCC14969	Illumina	15,603,988	100	15,603,366	100	SRR9192357
			pacBio	150,292	5,458	95,691	8,339	SRR9290549
4	*Streptomyces nitrosporeus*	ATCC12769	Illumina	15,858,551	100	15,857,973	100	SRR9192358
			pacBio	150,292	5,007	101,572	7,222	SRR9290548
5	*Streptomyces subrutilus*	ATCC27467	Illumina	23,157,475	100	23,157,337	100	SRR9192359
			pacBio	150,292	8,757	92,569	13,591	SRR9290547
6	*Streptomyces viridosporus T7A*	ATCC39115	Illumina	24,919,393	100	24,918,458	100	SRR9192360
			pacBio	150,292	7,910	92,139	12,571	SRR9290546
7	*Streptomyces kanamyceticus*	ATCC12853	Illumina	14,877,606	100	14,877,044	100	SRR9192361
			pacBio	150,292	5,044	59,627	12,291	SRR9290545
8	*Streptomyces aureofaciens*	ATCC11989	Illumina	19,155,832	100	19,155,718	100	SRR9192362
			pacBio	150,292	5,890	88,462	9,681	SRR9290544
9	*Streptomyces prasinus*	ATCC13879	Illumina	18,655,418	100	18,654,718	100	SRR9192388
			pacBio	150,292	5,560	84,107	9,288	SRR9290553
10	*Streptomyces fradiae*	ATCC10745	Illumina	9,446,513	100	9,445,832	100	SRR9192354
			pacBio	150,292	6,843	94,568	10,538	SRR9290561
11	*Streptomyces alboniger*	ATCC12461	Illumina	16,425,356	100	16,425,241	100	SRR9192355
			pacBio	300,584	3,638	159,270	6,351	SRR9290560
12	*Streptomyces coeruleorubidus*	ATCC13740	Illumina	17,546,981	100	17,546,866	100	SRR9192339
			pacBio	300,584	2,836	121,377	6,164	SRR9290563
13	*Streptomyces cinereoruber*	ATCC19740	Illumina	20,182,754	100	20,181,890	100	SRR9192340
			pacBio	150,292	3,319	52,279	9,266	SRR9290562
14	*Streptomyces nodosus*	ATCC14899	Illumina	14,034,927	100	14,034,843	100	SRR9192341
			pacBio	150,292	8,387	94,719	12,692	SRR9290557
15	*Streptomyces vinaceus*	ATCC27476	Illumina	6,612,160	100	6,610,219	100	SRR9192335
			pacBio	150,292	4,984	75,928	9,432	SRR9290559
16	*Streptomyces platensis*	ATCC23948	Illumina	14,819,357	100	14,819,243	100	SRR9192336
			pacBio	150,292	4,869	72,573	9,619	SRR9290558
17	*Streptomyces spectabilis*	ATCC27465	Illumina	18,717,018	100	18,716,280	100	SRR9192337
			pacBio	300,584	10,049	202,966	14,047	SRR9290555
18	*Streptomyces chartreusis*	ATCC14922	Illumina	16,988,966	100	16,988,850	100	SRR9192338
			pacBio	150,292	8,813	88,869	14,106	SRR9290554
19	*Stretpomyces rimosus*	ATCC10970	Illumina	25,109,758	100	25,108,764	100	SRR9192333
			pacBio	300,584	7,066	164,891	12,342	SRR9290564
20	*Streptomyces albofaciens*	ATCC23873	Illumina	19,360,703	100	19,360,564	100	SRR9192364
			pacBio	150,292	11,091	110,521	14,419	SRR9290552
21	*Streptomyces filamentosus*	ATCC23958	Illumina	21,181,460	100	21,180,615	100	SRR9192342
			pacBio	150,292	4,805	70,677	8,178	SRR9290556
22	*Streptomyces venezuelae* ATCC 10712	ATCC 10712	Illumina	18,667,122	100	18,664,663	100	SRR9192374
			pacBio	150,292	9,101	87,860	15,328	SRR9290565
23	*Streptomyces venezuelae* ATCC 21113	ATCC 21113	Illumina	21,561,533	100	21,559,491	100	SRR9192334
			pacBio	150,292	10,148	104,695	14,180	SRR9290566
24	*Streptomyces venezuelae* ATCC 10595	ATCC 10595	Illumina	16,798,923	100	16,797,236	100	SRR9192369
			pacBio	150,292	7,829	92,681	12,197	SRR9290567
25	*Streptomyces venezuelae* ATCC 15068	ATCC 15068	Illumina	15,310,620	100	15,308,905	100	SRR9192368
			pacBio	150,292	10,754	107,412	14,515	SRR9290568
26	*Streptomyces venezuelae* ATCC 14583	ATCC 14583	Illumina	19,423,668	100	19,421,332	100	SRR9192371
			pacBio	150,292	10,888	106,813	14,647	SRR9290569
27	*Streptomyces venezuelae* ATCC 14584	ATCC 14584	Illumina	15,447,783	100	15,446,240	100	SRR9192370
			pacBio	150,292	8,844	100,523	12,580	SRR9290540
28	*Streptomyces venezuelae* ATCC 14585	ATCC 14585	Illumina	51,795,644	100	51,791,331	100	SRR9192373
			pacBio	150,292	8,275	96,243	12,388	SRR9290541
29	*Streptomyces venezuelae* ATCC 21782	ATCC 21782	Illumina	19,569,337	100	19,567,069	100	SRR9192372
			pacBio	150,292	6,539	70,655	13,089	SRR9290542
30	*Streptomyces venezuelae* ATCC 21018	ATCC 21018	Illumina	16,469,406	100	16,467,790	100	SRR9192375
			pacBio	150,292	10,754	107,412	14,515	SRR9290543
